# Reference and point-of-care testing for G6PD deficiency: Blood disorder interference, contrived specimens, and fingerstick equivalence and precision

**DOI:** 10.1371/journal.pone.0257560

**Published:** 2021-09-20

**Authors:** Sampa Pal, Jane Myburgh, Pooja Bansil, Amanda Hann, Lynn Robertson, Emily Gerth-Guyette, Gwen Ambler, Greg Bizilj, Maria Kahn, Stephanie Zobrist, Michelle R. Manis, Nickolas A. Styke, Vajra Allan, Richard Ansbro, Tobi Akingbade, Andrew Bryan, Sean C. Murphy, James G. Kublin, Mark Layton, Gonzalo J. Domingo

**Affiliations:** 1 PATH, Seattle, Washington, United States of America; 2 Special Haematology Laboratory, Hammersmith Hospital, London, United Kingdom; 3 Departments of Laboratory Medicine and Microbiology, University of Washington School of Medicine, Seattle, Washington, United States of America; 4 Consultant, London, United Kingdom; 5 Vaccine and Infectious Disease Division, Fred Hutchinson Cancer Research Center, Seattle, Washington, United States of America; 6 Department of Laboratory Medicine and Pathology, University of Washington, Seattle, Washington, United States of America; 7 Center for Emerging and Re-emerging Infectious Diseases, University of Washington, Seattle, Washington, United States of America; Menzies School of Health Research: Charles Darwin University, AUSTRALIA

## Abstract

Certain clinical indications and treatments such as the use of rasburicase in cancer therapy and 8-aminoquinolines for *Plasmodium vivax* malaria treatment would benefit from a point-of-care test for glucose-6-phosphate dehydrogenase (G6PD) deficiency. Three studies were conducted to evaluate the performance of one such test: the STANDARD^™^ G6PD Test (SD BIOSENSOR, South Korea). First, biological interference on the test performance was evaluated in specimens with common blood disorders, including high white blood cell (WBC) counts. Second, the test precision on fingerstick specimens was evaluated against five individuals of each, deficient, intermediate, and normal G6PD activity status. Third, clinical performance of the test was evaluated at three point-of-care settings in the United States. The test performed equivalently to the reference assay in specimens with common blood disorders. High WBC count blood samples resulted in overestimation of G6PD activity in both the reference assay and the STANDARD G6PD Test. The STANDARD G6PD Test showed good precision on multiple fingerstick specimens from the same individual. The same G6PD threshold values (U/g Hb) were applied for a semiquantitative interpretation for fingerstick- and venous-derived results. The sensitivity/specificity values (95% confidence intervals) for the test for G6PD deficiency were 100 (92.3–100.0)/97 (95.2–98.2) and 100 (95.7–100.0)/97.4 (95.7–98.5) for venous and capillary specimens, respectively. The same values for females with intermediate (> 30% to ≤ 70%) G6PD activity were 94.1 (71.3–99.9)/88.2 (83.9–91.7) and 82.4 (56.6–96.2)/87.6(83.3–91.2) for venous and capillary specimens, respectively. The STANDARD G6PD Test enables point-of-care testing for G6PD deficiency.

## Introduction

Glucose-6-phosphate dehydrogenase (G6PD) (EC 1.1.1.49) is an essential enzyme in the pentose phosphate metabolic pathway. It helps maintain the levels of the co-enzyme nicotinamide adenine dinucleotide phosphate, and through this, glutathione. Glutathione plays an important role in red blood cell protection against oxidative challenges [1, 2]. The X-linked G6PD gene is highly polymorphic with several missense mutations, resulting in enzyme with reduced activity or more often reduced stability. These mutations result in higher proportions of red blood cells with suboptimal amounts of the enzyme and thus more susceptibility to lysis when exposed to an oxidative challenge [1, 2]. An estimated 400 million people have G6PD deficiency, a large proportion of whom reside in malaria-endemic regions [2, 3].

G6PD deficiency can manifest early at birth in the form of jaundice and is a significant risk factor for hyperbilirubinemia and in extreme cases permanent neurological damage (kernicterus) [4]. Later in life, exposure to certain bacterial and viral infections; foods such as favas; and several drugs, including rasburicase, dapsone, and the 8-aminoquinoline antimalarials primaquine and tafenoquine [2, 5–7], can trigger hemolytic events that sometimes require blood transfusion. Further, there is increasing evidence that SARS-CoV-2 enhances susceptibility to hemolytic triggers in G6PD deficient individuals [8–10], and conversely that G6PD deficiency is a predisposing factor for complications from COVID-19 [11, 12].

The most widely used assays to diagnose G6PD deficiency range from moderately complex, such as the fluorescent spot test (FST), to highly complex, such as the spectrophotometric quantitative reference assay [13]. While able to accurately identify G6PD deficiency, the FST is less accurate in discriminating females with intermediate G6PD activity who may be heterozygous, with one normal and one deficient G6PD gene allele [13]. The spectrophotometric reference assay is considered the gold standard for clinical diagnosis, and while primarily conducted at clinical reference laboratories and research laboratories, still suffers from interlaboratory variability and challenges in diagnosing females with intermediate G6PD activity [13–15]. Neither the FST nor the reference assay is appropriate for use at the point of care. In practice, the results from these tests are not available until at least 24 hours after request and often longer. Point-of-care tests for G6PD deficiency could improve case management both for newborns and to inform therapeutic decisions as indicated by cost-effectiveness studies [16–18].

Until recently, the only point-of-care tests available were two qualitative tests; one is no longer available, and the second has seen limited uptake, in part due to the restricted operating conditions allowed by the instructions for use [19, 20]. Quantitative point-of-care tests for G6PD deficiency are emerging, but the feasibility of their use at the point of care and with fingerstick specimens has not been fully demonstrated [21–23]. Separately, the compatibility of fingerstick ethylenediaminetetraacetic acid dipotassium salt dihydrate (K_2_EDTA) blood for measuring G6PD activity has been demonstrated but not for non-anticoagulated fingerstick samples and not with a point-of-care test [24]. This article describes three studies designed to evaluate the (i) performance of a point-of-care test against blood specimens with multiple blood disorders; (ii) precision of the test on multiple G6PD finger replicates; and (iii) diagnostic performance of the test on both fingerstick and venous specimens across three point-of-care settings including laboratory settings for venous blood testing (Table 1). Contrived venous blood specimens covering the critical medical decision limits (MDLs: 30% and 70% G6PD activity) were generated through heat abrogation to support the diagnostic performance evaluation.

**Table 1:** Summary of studies conducted to support evaluation of the STANDARD G6PD Test.

Study purpose	Study site for the STANDARD G6PD Test	Reference testing site
Evaluate the performance of a point-of-care test against blood specimens with multiple blood disorders	Haematology laboratory at the Hammersmith Hospital, London, UK	Hammersmith Hospital Haematology laboratory
Evaluate the precision of the test on multiple G6PD fingerstick replicates	Biological Specialty Company blood donation center, Reading and Allentown, Pennsylvania, USA	UWMC-NW clinical laboratory, University of Washington Medical Center
Evaluate the diagnostic performance of the test on both fingerstick and venous specimens across three point-of-care settings	Prospective cross-sectional studies:	Studies a and d: PATH
	a. Plasma MedResearch, Boca Raton, Florida, USA	Studies b, c, and d: UWMC-NW clinical laboratory, University of Washington Medical Center, PATH
	b. Biological Specialty Company blood donation center, Reading and Allentown, Pennsylvania, USA	
	c. Fred Hutchinson Cancer Research Center Prevention Center, University of Washington, Seattle, Washington, USA	
	d. *Contrived specimen panel*. Laboratory Alliance of Central New York, LLC, Syracuse, New York, USA	

Abbreviations: G6PD, glucose-6-phosphate dehydrogenase; UWMC-NW, University of Washington Medical Center—Northwest clinical laboratory.

## Methods

### Human subjects

Four studies were conducted in which human specimens were utilized. Three of the studies were performed on adult volunteers in the United States to assess the performance of the STANDARD^™^ G6PD Test (SD BIOSENSOR, South Korea) on fresh fingerstick blood:

1. A prospective, cross-sectional diagnostic accuracy study recruiting adults presenting to the Plasma MedResearch clinical laboratory in Boca Raton, Florida, USA. Volunteers for this study were consented under a prospective blood collection study protocol (Western Institutional Review Board study number 20161665).
2. A prospective, cross-sectional diagnostic accuracy study recruiting all-comer adult participants at the Fred Hutchinson Cancer Research Center Prevention Center, University of Washington, Seattle, Washington, USA (ClinicalTrials.gov number NCT04010695; Fred Hutchinson Cancer Research Center institutional review board [IRB] study number 10091).
3. A prospective, cross-sectional diagnostic accuracy study recruiting adult African American blood donors at Biological Specialty Company’s blood donation center in Reading and Allentown, Pennsylvania, USA (ClinicalTrials.gov number NCT04033640; PATH IRB study number 1416844).

All US studies recruited healthy adults (older than 18 years of age), and all participants provided written informed consent.

The fourth study, to assess potential biological interferents for the STANDARD G6PD Test, used de-identified K_2_EDTA whole blood samples from requested testing already completed at the Hammersmith Hospital Haematology laboratory, London, United Kingdom, prior to the samples being discarded. The study was determined by the PATH Research Determination Committee as not requiring additional IRB approval.

None of the studies recorded personal identifiers, beyond, age, sex and race.

### Testing

#### Point-of-care testing for hemoglobin normalized G6PD activity

The STANDARD G6PD Test was used as per the instructions for use with either non-anticoagulated capillary blood collected from a fingerstick or with venous K_2_EDTA blood. The STANDARD G6PD Test provides a G6PD activity value normalized for hemoglobin concentration in units per gram hemoglobin (U/g Hb) and a value for the hemoglobin concentration provided in grams per deciliter. Briefly, the STANDARD G6PD is a point-of-care test for G6PD deficiency indicated for use on capillary fingerstick samples as well as venous K_2_EDTA samples. The kit provides disposable blood transfer tubes (STANDARD Ezi tube) to transfer 10 mL of blood to a vial with extraction buffer; and after mixing, to then transfer 10 mL of this processed sample to the disposable test device, which is already inserted into the instrument. The instrument provides a result for G6PD activity and hemoglobin concentration after 2 minutes. In all studies except the blood disorder interference study, both venous and capillary samples were entirely handled with the STANDARD G6PD Test disposable blood transfer tubes. In the blood disorder study, the first step, involving introducing the blood to the extraction buffer, was performed using an analytical pipette.

#### Point-of-care measurement of hemoglobin

The HemoCue^®^ Hb 201+ System was used as per the instructions for use with either non-anticoagulated capillary blood collected from a fingerstick or on venous K_2_EDTA blood. The HemoCue system measures hemoglobin concentration in grams per deciliter.

#### Reference G6PD activity measurement

Reference testing for G6PD activity was performed at the PATH research laboratories in Seattle, Washington, USA, on venous K_2_EDTA blood for the Florida fingerstick study, and at the Haematology laboratory at Hammersmith Hospital for the blood disorder interference study. Both laboratories used the quantitative G6PD kit from Pointe Scientific (catalog number G7583), according to the manufacturer’s instructions. Normal, intermediate, and deficient controls from Analytical Control Systems, Inc. (catalog numbers HC-108, HC-108IN, and HC-108DE, respectively) were run using the same method on each day of testing. At the PATH laboratories, absorbance was measured on a Shimadzu UV-1800 six-cell, temperature-regulated spectrophotometer, and at Hammersmith Hospital on the Beckman Coulter DU^®^ 800 temperature-regulated spectrophotometer (serial number 8002840). All G6PD activity rates were normalized for hemoglobin concentration and presented as U/g Hb. All Pointe Scientific testing was performed at 37°C.

Reference testing for the clinical studies conducted at the Pennsylvania and Washington sites was conducted at the University of Washington Medical Center—Northwest (UWMC-NW) clinical laboratory, Seattle, Washington, USA, which is certified by the Clinical Laboratory Improvement Amendments (CLIA number 50D0633053). G6PD activity was measured with the Pointe-Scientific G6PD reagent kit on the automated Beckman Coulter AU680 clinical chemistry analyzer. The G6PD activity was normalized using the hemoglobin concentration determined in the same clinical laboratory.

#### Reference hemoglobin concentration measurement

The hemoglobin concentration values used to normalize the G6PD activity measurements for the final reference values were determined from the venous K_2_EDTA blood samples at both the PATH and Hammersmith Hospital sites. At PATH, they were determined with the HemoCue Hb 201+ System. At Hammersmith Hospital, they were measured as part of the routine clinical laboratory work-up, either with three Abbott Alinity^™^ full blood count analyzers or the Sysmex XT-2000*i*^™^, also a full blood count analyzer. For the clinical studies conducted at the Pennsylvania and Washington sites, hemoglobin concentration was determined on the Sysmex XN-2000 CBC instrument.

#### Fluorescent spot test

For a subset of the three clinical samples collected in the United States, the FST or Trinity Biotech G-6-PDH screening test was conducted at the PATH laboratories. The FST is a qualitative test performed by incubating a small amount of blood with glucose-6-phosphate and nicotinamide adenine dinucleotide phosphate. Drops of the mixture are removed at 5-minute intervals, spotted on filter paper, and then viewed under long-wave ultraviolet light. The FST was conducted and interpreted to classify individuals into deficient, intermediate, or normal categories by qualitative visual assessment of signal intensity, as per the manufacturer instructions.

#### Confirmatory assays for defining blood conditions

The blood specimens for the UK blood disorder interference study were tested for blood disorders as part of routine clinical practice, upon request from the clinic. S1 Table lists the confirmatory testing used to diagnose the blood disorders.

#### White blood cell depletion

For clinical specimens with a WBC count of > 30 x 10^9^ cells/L, the Hammersmith Haematology laboratory routinely submits the specimens to a WBC depletion protocol prior to measuring G6PD activity (described in S1 Text). For this study, the reference G6PD assay testing was performed on both the undepleted high WBC count clinical specimens and their matching specimens after WBC depletion. The STANDARD G6PD Test was also run on both the depleted and undepleted specimens.

### Blood disorder interference study

The Hammersmith Hospital Haematology laboratory, where the blood disorder interference study was conducted, is part of the North West London Pathology service, a National Health Service diagnostic laboratory. All samples used in this study were K_2_EDTA whole blood samples from requested testing that had already been completed, and were subsequently discarded. Samples were purposefully selected to collect as diverse a set of blood conditions as possible. Samples required for the study were identified through monitoring of results from the Haematology and Biochemistry Department during routine daily work. All samples were stored in the laboratory’s temperature-controlled refrigerators and were tested within one week (7 days) of collection, as per laboratory standard operating procedure. Some samples tested as part of this study were not blinded to G6PD status, as known G6PD deficient patients identified in the laboratory during the course of the study were then tested on the STANDARD G6PD Test.

### Fingerstick study specimen collection and testing

The workflows for the fingerstick studies are illustrated in S1 Fig. For the fingerstick study conducted at the Plasma MedResearch clinical laboratory, healthy adult participants were tested with the point-of-care STANDARD G6PD Test on the second drop of non-anticoagulated blood, and for capillary hemoglobin concentration with the HemoCue Hb 201+ System on the third drop of blood at the same study site. Venous blood in K_2_EDTA anticoagulant (5 mL) was collected from each study participant and sent on ice packs by overnight courier service within 24 hours of collection to the PATH research laboratories, where the reference G6PD assay was conducted as described above. The STANDARD G6PD Test was also run on a separate device at PATH on the venous K_2_EDTA blood specimens. All testing at PATH was performed within 48 hours of specimen collection. Laboratory staff at the Plasma MedResearch facility and at the PATH facility were blinded to the reference G6PD and hemoglobin values.

For the Pennsylvania and Washington clinical studies, healthy adult participants were tested with the point-of-care STANDARD G6PD Test on the second drop of non-anticoagulated blood, and for capillary hemoglobin concentration with the HemoCue Hb 201+ System on the third drop of blood. Two 5 mL vacutainers of venous blood in K_2_EDTA anticoagulant were also collected from each study participant. One was used to conduct the STANDARD G6PD Test on venous blood and the HemoCue Hb 201+ System at the site of collection, with the remainder sent to the PATH laboratories. The second vacutainer was sent to the UWMC-NW clinical laboratory for reference testing.

### Contrived specimen study

In addition, data are presented from an additional contrived specimen study. Contrived specimens were prepared at PATH to increase the number of specimens in the intermediate G6PD activity range and close to the critical thresholds or the clinical decision-making points. A panel of 90 contrived specimens, spanning the critical G6PD activity thresholds for G6PD deficient and intermediate activity as per World Health Organization (WHO) Prequalification of In Vitro Diagnostics Programme case definitions, was developed at the PATH laboratories by heat abrogation of G6PD activity using a method adapted from the UK National External Quality Assurance Services G6PD scheme [25]. Briefly, venous acid citrate dextrose whole blood specimens were obtained from blood donors who had provided written consent through the PlasmaLab Everett blood bank, Everett, Washington, USA. Specimens arrived at PATH within 4 hours of collection. An aliquot was refrigerated upon arrival and the remainder of the blood was incubated at 45°C in a water bath. Aliquots were then withdrawn at the following time intervals: 1, 2, 3, 4, and 5 hours. At 5 hours, all specimens were randomized and sent blinded, simultaneously, to the Laboratory Alliance of Central New York, LLC (Syracuse, New York, USA) to perform the STANDARD G6PD Test, and to the UWMC-NW clinical laboratory to perform the reference testing for G6PD activity. Additionally, and simultaneously PATH performed the reference testing for G6PD activity on the same samples. Overall, 15 clinical specimens were used to generate a panel of 90 contrived specimens over 3 days, with 5 specimens undergoing heat abrogation each day. Additionally, five contrived specimens spanning a broad hemoglobin concentration range were developed by plasma level adjustment. The entire 95 randomized samples were created over three batches and all testing was completed within 24 hours of sample generation. The workflow for the contrived specimen study is presented in S2 Fig.

### Fingerstick precision study

A fingerstick precision study was performed at the Pennsylvania site to assess the precision of the STANDARD G6PD Test on non-anticoagulated blood. Samples were tested as they were drawn, utilizing two analyzers and two operators so that each duplicate from a single finger could be tested immediately.

Participants were also enrolled in the Pennsylvania cross-sectional clinical study, and their first test results from the clinical study using the STANDARD G6PD Test were used to screen for eligibility for the capillary precision study. Fifteen participants were recruited: five males with G6PD values < 3.0 U/g Hb (Level 1); five females (intermediates) with G6PD values of 3.0 to 7.0 U/g Hb (Level 2); and three males and two females with G6PD values > 7.0 U/g Hb (Level 3) as measured on the STANDARD G6PD Test.

Once a participant was enrolled in the study, fingerstick capillary samples were collected and tested from two fingers on one hand and two fingers on the second hand in accordance with the instrument instructions for use, generating 8 measurements of G6PD activity per participant and a total of 120 measurements.

### G6PD gene sequencing

A limited number of specimens collected in the United States, primarily covering the critical G6PD activity dynamic range, were selected for full G6PD gene sequencing. Not all study participants provided consent for sequencing. The full G6PD gene was sequenced by Fulgent Genetics (El Monte, California, USA), a third-party laboratory CLIA qualified to perform high-complexity testing (CLIA number 05D2043189), through next-generation sequencing. A minimum of 10x coverage was obtained for all G6PD exons. A total of 35 samples were sequenced, all of which were from individuals who provided consent for this testing.

### Statistical analysis

The performance of the STANDARD G6PD Test against the spectrophotometric reference test was determined by calculating the test’s sensitivity and specificity as described previously [26]. Absolute G6PD values on the reference assay were normalized at each site to facilitate interlaboratory comparison. Reference values were expressed as the percentage of each site’s adjusted male median determined from 36 randomly selected G6PD normal males who were subsequently excluded from the performance analysis [27, 28].

Case definitions for evaluating STANDARD G6PD Test performance, in terms of G6PD status results, are presented according to the clinically relevant thresholds used to inform the administration of 8-aminoquinolines: G6PD deficient cases were defined as males and females with ≤ 30% G6PD activity, and females with intermediate G6PD activity were defined as those with > 30% activity and ≤ 70% activity. Case definitions for evaluating STANDARD G6PD Test performance, in terms of hemoglobin status, are based on the WHO case definitions [29].

All statistical analyses were performed using Stata^®^ 15.0 (StataCorp).

## Results

### Blood donor demographics

The sex and age breakdowns of the participants in all studies are provided in Table 2. For the fingerstick studies conducted in the United States, recruitment focused primarily on African Americans to maximize the probability of recruiting individuals with G6PD deficiency. Ethnic origins were not available for the blood disorder study in the United Kingdom. Participants were recruited at the Plasma MedResearch clinical laboratory for the fingerstick study in Florida, USA, over the months July to September 2018; and for the blood disorder study, blood specimens were tested between March 5 and July 3 2019. The studies in Washington and Pennsylvania were conducted from May to October 2019 and September 2019 to January 2020, respectively.

**Table 2:** Study donor demographics by age, sex, race, G6PD deficiency status, and anemia as determined by hemoglobin concentration. The demographics are provided for the total number of study participants included in the analysis.

	London, United Kingdom	Florida, USA	Washington, USA	Pennsylvania, USA	Combined
**Final analytic population, n**	167	189	213	221	790
**Age (years)**					
Mean (standard deviation)	48.1 (23.8)	38.4 (14.8)	35.4 (12.3)	37.5 (12.5)	39.4 (16.6)
Range	2 days–94 years	18–79 years	19–65 years	18–65 years	2 days–94 years
**Age categories, n (%)**					
Neonates (< 1 month)	6 (3.4)	—	—	—	6 (0.8)
Infants (1 month to < 2 years)	11 (6.6)	—	—	—	11 (1.4)
2–11 years	1 (0.6)	—	—	—	1 (0.1)
12–15 years	1 (0.6)	—	—	—	1 (0.2)
16–64 years	103 (61.7)	181 (95.8)	220 (99.5)	210 (98.6)	714 (90.4)
65+ years	45 (27.0)	8 (4.2)	1 (0.5)	3 (1.4)	57 (7.2)
**Sex, n (%)**					
Female	87 (52.1)	116 (61.4)	135 (63.4)	58 (26.2)	396 (50.1)
Male	80 (47.9)	73 (38.6)	78 (36.9)	163 (73.8)	394 (49.9)
**Race, n (%)**					
Asian	—	—	27 (12.7)	—	27 (3.4)
Black/African American	—	189 (100.0)	34 (16.0)	221 (100.0)	444 (56.2)
Caucasian/White	—	—	133 (62.4)	—	133 (16.8)
More than one race	—	—	15 (7.0)	—	15 (1.9)
Other	—	—	4 (1.9)	—	4 (0.5)
Not reported	167 (100.0)	—	—	—	167 (21.1)
**G6PD status, n (%)**					
Deficient < 30%	10 (6)	23 (12.2)	7 (3.3)	16 (7.2)	56 (7.1)
Intermediate 30–70%	10 (6)	7 (3.7)	4 (1.9)	6 (2.7)	27 (3.4)
Intermediate 70–80%	3 (1.8)	6 (3.2)	4 (1.9)	5 (2.3)	18 (2.3)
Normal > 80%	144 (86.2)	153 (81.0)	198 (93.0)	194 (87.8)	689 (87.2)
**Anemia status**,^‡^**n (%)**					
Non/mild	99 (59.3)	159 (84.1)	209 (98.1)	201 (91.0)	668 (84.6)
Moderate	54 (32.3)	29 (15.3)	3 (1.4)	20 (9.0)	106 (13.4)
Severe	14 (8.4)	1 (0.5)	1 (1.4)	—	16 (2.0)

Abbreviation: G6PD, glucose-6-phosphate dehydrogenase.

^‡^ For the Washington and Pennsylvania (USA) sites, determined using CBC.

For the United Kingdom and Florida (USA) sites, determined using the HemoCue Hb 201+ System. Anemia classification was age and sex adjusted.

### Point-of-care test users

The data presented in this study were collected over at least 15 STANDARD G6PD instruments and more than six reagent kit lots. The users included one expert hematology laboratory staff member at the UK facility, and a medical doctor, nurses, phlebotomists, certified medical technologists, and other professional laboratory staff at the US clinical study sites.

### Effect of blood disorders on the performance of the STANDARD G6PD Test

**Fig 1:**
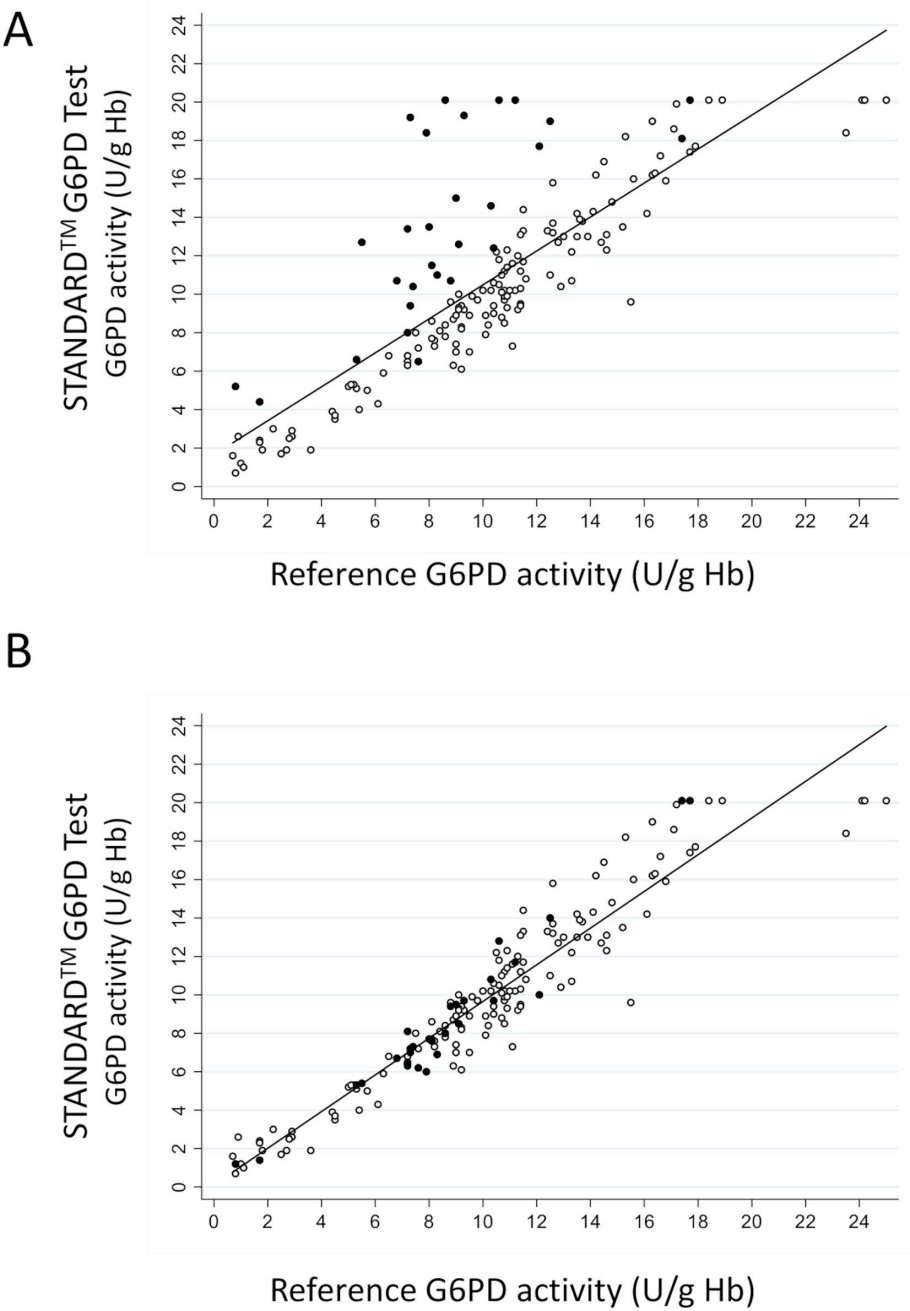
Correlation between the STANDARD G6PD Test result and that of the reference assay as performed in the clinical laboratory. Specimens with a white blood cell (WBC) count of > 30 x 10^9^ cells/L are represented by the solid black circles. All other study samples are indicated by the open circles. The clinical laboratory performs WBC depletion on all specimens with WBC counts > 30 x 10^9^ cells/L. (A) Linear regression plot for the STANDARD G6PD Test run on whole blood and the reference assay run on whole blood or depleted blood according to laboratory protocols. (B) Linear regression plot for the STANDARD G6PD Test and the reference assay, both run on whole or depleted blood according to laboratory protocols.

The results of the STANDARD G6PD Test were compared to those of the reference assay across a panel of blood specimens with a range of blood disorders and other potential endogenous interferents (Table 3). The effect on blood specimens with high WBC counts were considered separately (described below). A total of 167 specimens were tested from donors spanning all ages, from neonates to adults. Many samples fell into more than one category of disorder, such that some specimens fit into two or three blood disorder categories; these are listed as secondary and tertiary conditions. There were at least five representative cases for all disorders, with the exception of spherocytosis and hemoglobin D. A linear regression between the STANDARD G6PD Test results and the reference assay showed a high degree of correlation, with an R-squared value of 0.90 (Fig 1A and 1B). The STANDARD G6PD Test also provides a hemoglobin result. A linear regression fit of the correlation between the STANDARD G6PD hemoglobin concentration and the hemoglobin measured on the CBC analyzers showed an R-squared value of 0.95 (S3 Fig). Both the G6PD values and the hemoglobin values showed good agreement across the dynamic range compared to the reference assays in this study (Fig 1 and S3 and S4 Figs).

**Table 3:** Inventory of blood specimens and laboratory-confirmed blood disorders and potential endogenous interfering substances for the 167 specimens tested in the study performed at Hammersmith Hospital, United Kingdom. Multiple disorders were associated with some specimens and were counted as secondary and tertiary samples in the table.

	Number			Total
	Primary	Secondary	Tertiary	
Hyperbilirubinemia	7	4	2	13
Hyperproteinemia (globulin result)	5	6	1	12
Hyperglycemia	5	0	2	7
Raised lactic acid	4	1	0	5
Raised lactate dehydrogenase	0	9	4	13
Raised hematocrit	6	1	0	7
Low hematocrit	3	3	6	12
Raised retics	4	9	1	14
Raised white blood cell count	28	1	0	29
Raised platelets	7	3	1	11
Sickle hemoglobin (HbAS, HbSS, HbSC)	14	1	2	17
Hemoglobin D	4	0	0	4
Hemoglobin E	8	0	0	8
Hemoglobin C	4	3	0	7
Alpha thalassemia	5	1	0	6
Beta thalassemia (A2%)	6	0	0	6
Iron deficiency	5	3	1	9
Renal diagnostic (creatinine)	5	4	2	11
Spherocytosis	1	0	0	1
Platelet clumps (not clotted)	5	0	0	5
Raised C-reactive protein	6	9	0	15
Raised cholesterol	4	1	0	5
Elevated lipids (triglycerides)	5	2	1	8
Copper-containing compounds	5	0	0	5
Other	21	0	0	21
**Total**	**167**			

A semiquantitative interpretation of the G6PD results using the current thresholds on the STANDARD G6PD Test of 4.0 U/g Hb and 6.0 U/g Hb for deficient and intermediate, respectively, resulted in an agreement of 98.2% (95% confidence interval [CI]: 87.1–96.5) across all specimens (S2 Table). A semiquantitative interpretation of the hemoglobin results using the WHO classifications [29] resulted in an agreement of 88% (95% CI: 82.1–92.5) on all specimens (S3 Table).

### Impact of high white blood cell count on whole blood G6PD activity

A total of 28 blood specimens with WBC counts of > 30 x 10^9^ cells/L were included in the blood disorder study. As per the laboratory protocols, these samples underwent WBC depletion prior to G6PD testing on the reference assay. For purposes of this study, the pre-depleted and depleted blood specimens were tested on both the STANDARD G6PD Test and the reference assay. The descriptive statistics are provided in S4 Table. Briefly, the mean WBC count was reduced from 77.4 x 10^9^ cells/L to 9.3 x 10^9^ cells/L by the depletion step. The mean G6PD activity for the 28 specimens dropped by 4.2 U/g Hb on depletion on the reference assay and 4.9 U/g Hb on the STANDARD G6PD Test. Both tests would have misclassified the same two G6PD deficient specimens as G6PD normal in the pre-depleted blood. Fig 1A shows the pre-depleted G6PD activity on the STANDARD G6PD Test for the 28 high WBC count specimens compared to the respective depleted G6PD activity on the reference assay, and Fig 1B shows the depleted STANDARD G6PD Test G6PD activity results against the same depleted reference assay results. The STANDARD G6PD Test behaved equivalently to the reference assay for G6PD activity in specimens with extremely high WBC counts.

### Contrived specimen panel

A 95-member panel of contrived whole blood specimens designed to span the MDLs from just < 30% G6PD activity to just > 80% G6PD activity, as well as the dynamic hemoglobin range, was created to evaluate the STANDARD G6PD Test. These data were intended to supplement for samples in the intermediate G6PD activity range, which are hard to recruit. Aliquots of the same panel member were sent blinded to the Laboratory Alliance of Central New York for testing on the STANDARD G6PD Test, as well as to the UWMC-NW clinical laboratory in Washington, USA, for reference testing on an automated blood analyzer. A third aliquot was tested at the PATH laboratories on a temperature-controlled spectrophotometer.

**Fig 2:**
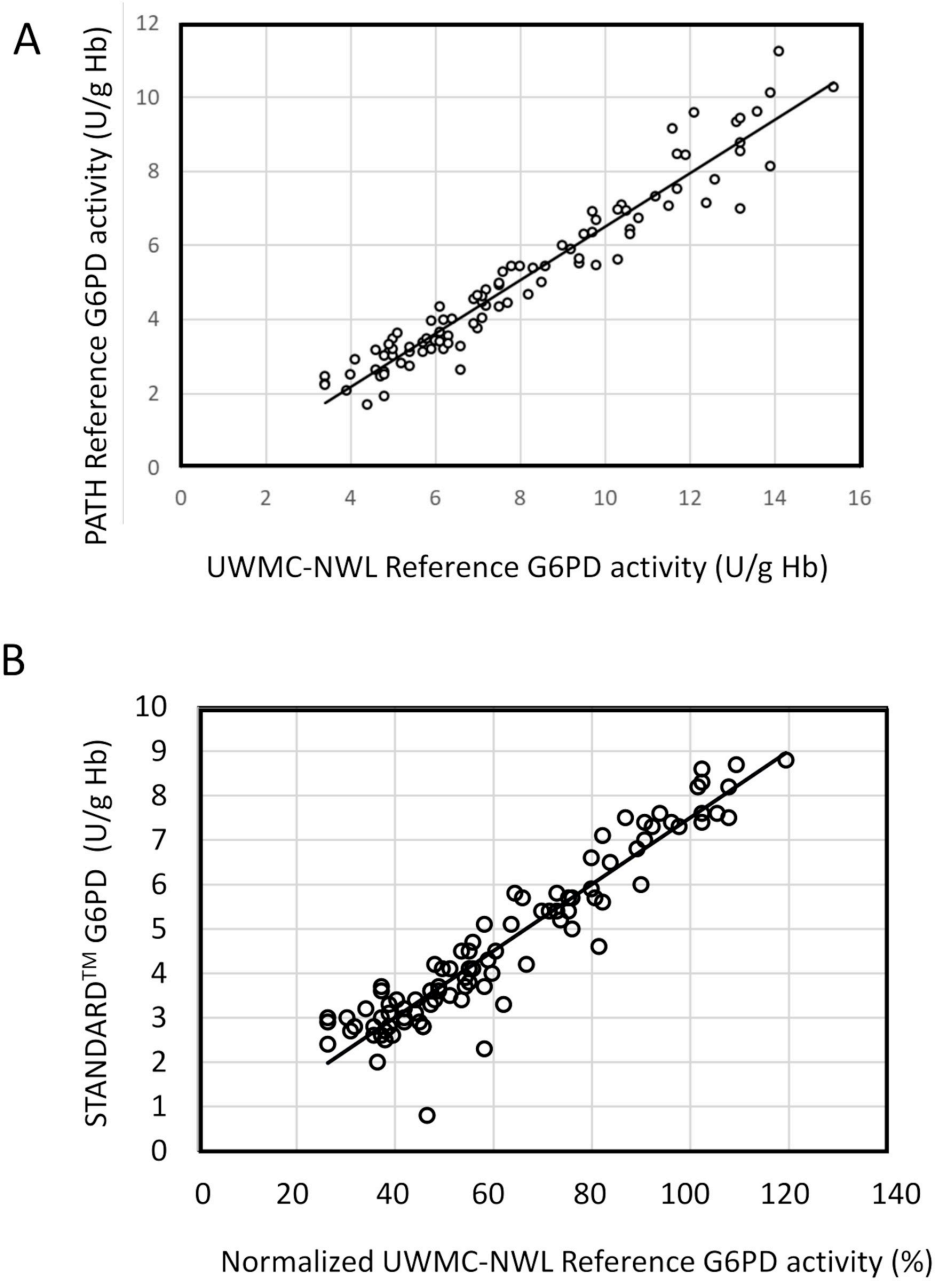
Linear correlations for STANDARD G6PD Test and reference G6PD values on contrived specimens spanning the medical decision limits. (A) Correlation between the Pointe Scientific reagent kit run on a spectrophotometer at one laboratory (PATH, Washington, USA) and on an automated clinical chemistry analyzer at a second laboratory (University of Washington Medical Center—Northwest clinical laboratory [UWMC-NW], Washington, USA). (B) Correlation between the STANDARD G6PD Test and normalized (to percent G6PD activity) UWMC-NW laboratory reference G6PD values. Solid lines represent the linear regression fits.

The reference assays conducted at PATH and the UWMC-NW clinical laboratory were compared. Comparison of the reference G6PD activity for the panels showed good correlation, with an R-squared value of 0.93, but poor agreement in terms of absolute G6PD activity values (Fig 2A). The G6PD activity values were significantly higher at the UWMC-NW clinical laboratory with a mean value of 8.0 U/g Hb (95% confidence interval: 7.4–8.6) versus the PATH values with a mean of 5.1 U/g Hb (95% confidence interval: 4.6–5.6). Normalizing each reference assay to percent activity resulted in a linear regression with a gradient of 1.2 (S5 Fig), highlighting the limitations of normalization of G6PD activity. The STANDARD G6PD Test showed good linear correlation against the reference assay results from the UWMC-NW clinical laboratory across the critical MDL dynamic range, with an R-squared value of 0.90 (Fig 2B). The mean G6PD activity for specimens with ≤ 3.0, 3.1 to 6.0, and > 6.0 U/g Hb on the STANDARD G6PD Test were 39%, 58%, and 97%, respectively. All specimens with ≤ 70% G6PD activity had < 6.0 U/g Hb activity on the point-of-care test.

### Fingerstick precision

**Fig 3:**
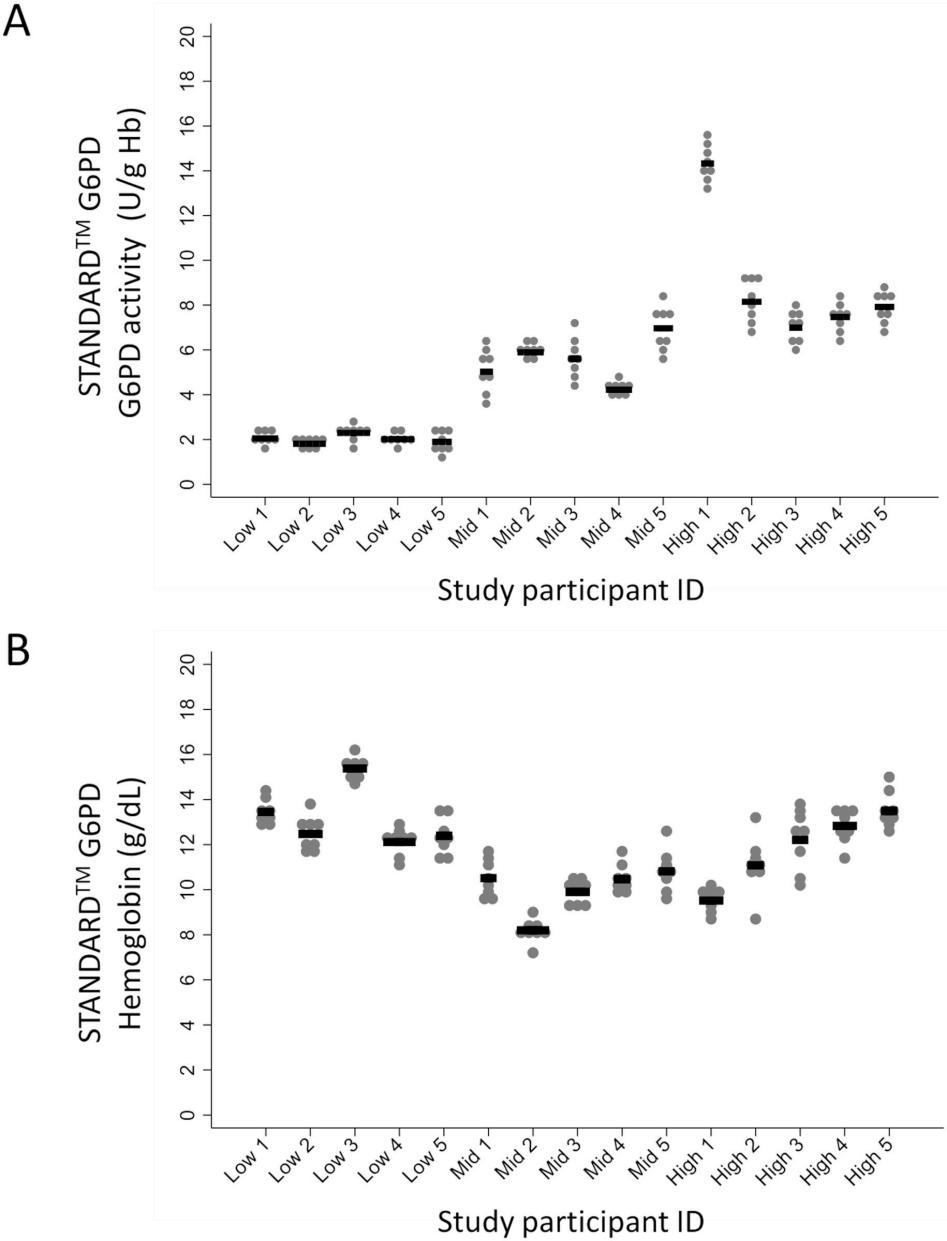
Fingerstick precision for the STANDARD G6PD Test. Fifteen study participants with G6PD deficient, intermediate, and normal activity on the STANDARD G6PD Test were tested on both hands by two operators across four fingers (two per hand), for a total of eight STANDARD G6PD Test runs. (A) STANDARD G6PD Test results per study participant. (B) Hemoglobin test results per study participant.

A fingerstick precision study was conducted to investigate the variance in G6PD and hemoglobin measurements from multiple fingerstick samples, nested in the cross-sectional Pennsylvania study. In order to span the MDL dynamic range, five participants with each of the following ranges of G6PD activity on the STANDARD G6PD Test were recruited into the fingerstick precision study: (i) < 3.0 Ug/Hb; (ii) 3.0 to 7.0 U/g Hb; and (iii) >7.0 U/g Hb. For each study participant, two fingersticks per hand were tested in duplicate across two instruments and two operators, for a total of 8 replicate fingerstick STANDARD G6PD Test results per participant and in total 120 test results. (Fig 3A).

For G6PD testing, the samples provided repeatable results from multiple fingersticks (Fig 3A). For samples with G6PD activity ≥ 3.0 U/g Hb the mean coefficient of variance was 10.3% with two individuals exceeding 15%, for specimens with G6PD activity < 3.0 U/g Hb the standard deviation was less than 0.5 U/g Hb. The results (n = 40) for all five deficient participants consistently fell below the threshold for G6PD deficiency (< 4.0 U/g Hb). Only 1 of 40 results for normal patients (G6PD > 6.0 U/g Hb) was discordant, at 5.7 U/g Hb, from an average of 7.0 U/g Hb. For male patients, this result would still be classified as normal. All other discrepancies were from the four intermediate participants (4.0 to 6.0 U/g Hb). Two results from one participant were < 4.0 U/g Hb, where the average result was 5.0, and four results from two participants exceeded 6.0 U/g Hb, where the averages were 5.7 and 5.8. The overall percent agreement for correctly classifying across deficient, intermediate, and normal cases was 94.2% (95% CI: 88.4–97.6). These results support the precision of the STANDARD G6PD Test on capillary specimens when reporting G6PD results as semiquantitative.

For 11 of the 15 study participants, the coefficient of variance was ≤ 7% in the hemoglobin concentration. For two participants, variance was higher than expected (Fig 3B).

### Clinical performance

**Fig 4:**
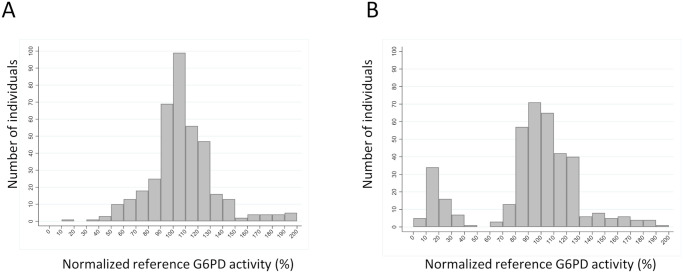
Histogram representation of the G6PD activity distributions in (A) females and (B) males. The reference G6PD values (U/g Hb) were normalized using the male medians and are presented here in percent activity.

The performance of the STANDARD G6PD Test on non-anticoagulated fingerstick blood was evaluated across three prospective clinical studies at three different clinics in the United States. Fig 4 shows the G6PD distribution across all studies for males and females using a percent G6PD activity scale by site-specific normalization to a male median G6PD value.

**Fig 5:**
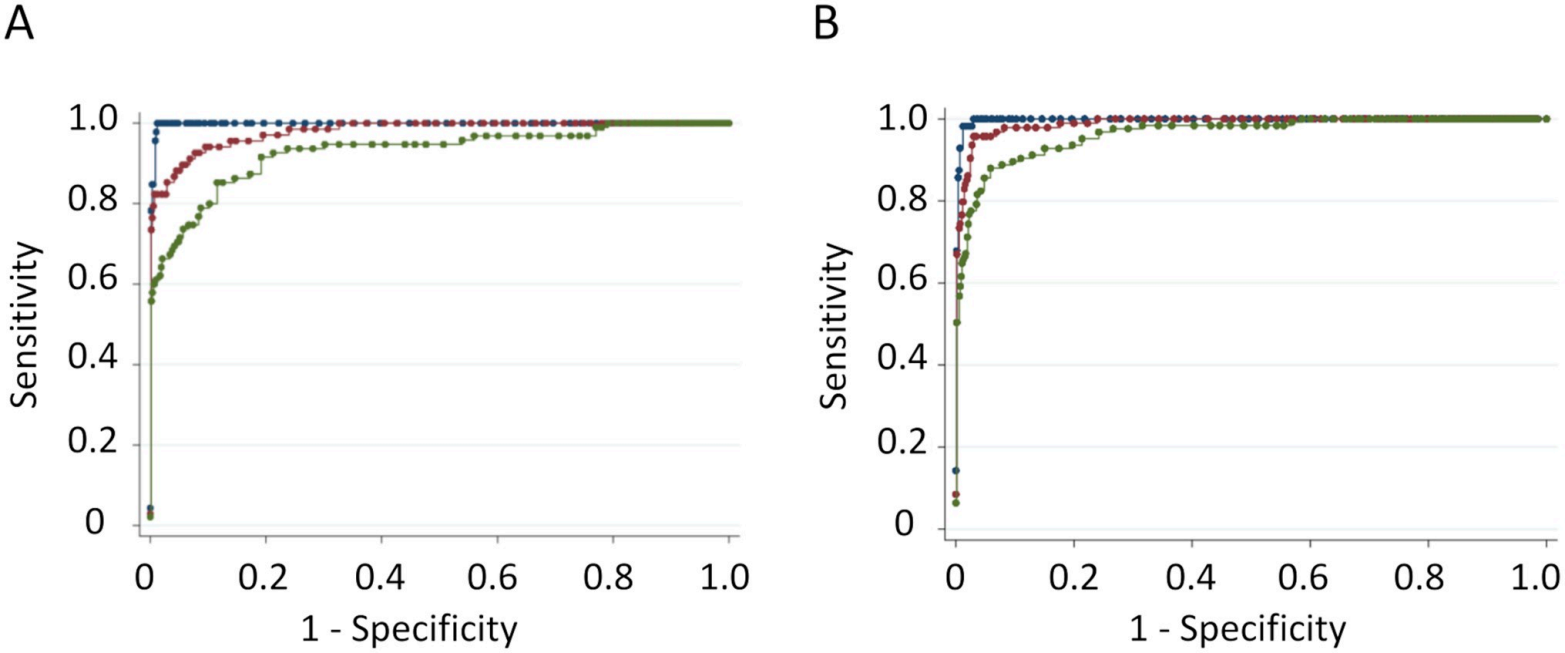
Receiver operating characteristics for the STANDARD G6PD Test against the reference assays for G6PD in capillary specimens. Discrimination of G6PD deficient males and females (≤ 30% G6PD activity) from females with intermediate activity and males and females with normal activity (dark blue); discrimination of females with intermediate activity (> 30% and ≤ 70% G6PD activity) from females with normal G6PD activity (red); and discrimination of females with intermediate activity (> 30% and ≤ 80% G6PD activity) from females with normal G6PD activity. (green), for (A) capillary specimens and (B) venous specimens.

The ability of the point-of-care test to discriminate deficient, intermediate, and normal individuals was assessed through receiver operating characteristic (ROC) and area under the curve (AUC) analysis (Fig 5 for (A) capillary and (B) venous samples). In addition to males and female deficient (≤ 30%. G6PD activity), ROC curves are shown for two G6PD intermediate ranges: (i) > 30% to ≤ 70%, and (ii) > 30% to ≤ 80%. For the intermediate ROC curves, only females with > 30% activity were analyzed. The AUCs for deficient males and females (≤ 30% G6PD activity), intermediate females (> 30% to ≤ 70% G6PD activity), and intermediate females (> 30% to ≤ 80% G6PD activity) on capillary specimens were 0.997, 0.942, and 0.891, respectively, and on venous specimens were 0.997, 0.976, and 0.945, respectively.

The sensitivity and specificity of the STANDARD G6PD Test for G6PD deficient males and females and for females with intermediate G6PD activity is summarized in Table 4. The same 6.0 U/g Hb threshold on the STANDARD G6PD was used for females with intermediate G6PD activity at the 70% and 80% thresholds. The STANDARD G6PD Test showed equal performance for individuals with severe G6PD activity (≤ 30%) across both venous and capillary specimens. The performance dropped for intermediates from venous to capillary; although at the 70% threshold, the confidence intervals for both venous and capillary specimens overlapped.

**Table 4:** Diagnostic performance of the STANDARD G6PD Test using the manufacturer’s thresholds compared with normalized spectrophotometric reference values, by specimen type.

	Venous	Capillary
30% G6PD deficient males and females, total study number N_D_		
Sensitivity (95% CI)	100.0	100.0
	(92.3–100.0)	(92.3–100.0)
Specificity (95% CI)	97.0	97.4
	(95.2–98.2)	(95.7–98.5)
70% G6PD intermediate females, total study number N_I_		
Sensitivity (95% CI)	94.1	82.4
	(71.3–99.9)	(56.6–96.2)
Specificity (95% CI)	88.2	87.6
	(83.9–91.7)	(83.3–91.2)
80% G6PD intermediate females, total study number N_I_		
Sensitivity (95% CI)	84.4	71.9
	(67.2–94.7)	(53.3–86.3)
Specificity (95% CI)	91.6	90.2
	(87.7–94.6)	(86.1–93.5)

Abbreviations: CI, confidence interval; G6PD, glucose-6-phosphate dehydrogenase.

N_I_, total sample size for G6PD intermediate performance (all females, not including G6PD deficient females). N_D_, total sample size for G6PD deficiency (both males and females).

It is important to assess the percent G6PD activity of false negatives at the intermediate activity level, since the risk of hemolysis decreases with increasing percent G6PD activity. This is summarized in Table 5 in terms of percent positivity for females with less than 70%, 65%, and 60% activity at the 6.0 U/g Hb threshold. There were three false negative results, all of which were on specimens with G6PD activity > 60% on the reference assay.

**Table 5:** Diagnostic performance of the 6.0 U/g Hb threshold on the STANDARD G6PD Test for females with G6PD deficient and intermediate activity on capillary specimens. The percent positivity, negativity, and predictive power for all females with > 70%, 65%, and 60% activity are shown. Ninety-five percent confidence intervals are provided in brackets.

	Reference G6PD activity		
	70%	65%	60%
**Percent positivity**	83.3% (58.6–96.4)	92.3% (64.0–99.8)	100% (63.1–100.0)
**Percent negativity**	87.6% (83.3–91.2)	86.8% (82.4–90.5)	85.7% (81.2–89.5)
**Positive predictive power**	29.4% (17.5–43.8)	23.5% (12.8–37.5)	15.7% (7.0–28.6)
**Negative predictive power**	98.8% (96.6–99.8)	99.6% (97.9–100.0)	100% (98.6–100.0)

Abbreviation: G6PD, glucose-6-phosphate dehydrogenase.

The ability of the STANDARD G6PD Test to discriminate G6PD deficient males and females, females with intermediate G6PD activity (> 30 to ≤ 70%), and normal females and males was assessed in terms of percent agreement (Table 6). The percent agreement for venous samples is provided in the supplemental files. The total percent agreement for capillary specimens was 92% (95% CI: 89.5–94.0).

**Table 6:** Percent agreement between the STANDARD G6PD Test and the spectrophotometric reference test using the manufacturer’s threshold values at 30% and 70% G6PD activity thresholds, on capillary specimens.

		Spectrophotometric reference test			Total
		Deficient	Intermediate	Normal	
**STANDARD G6PD Test**	**Deficient**	46	8	7	61
	**Intermediate**	0	6	32	38
	**Normal**	0	3	520	523
	**Total**	46	17	559	622

Abbreviation: G6PD, glucose-6-phosphate dehydrogenase.

Linear regression of the STANDARD G6PD Test’s G6PD activity measurements as compared to the normalized reference assay results showed an R-squared correlation value of 0.65 for both venous and capillary specimens (S6 Fig).

Correlation of the hemoglobin results for capillary and venous STANDARD G6PD Test measurements against the reference hemoglobin value were R-squared = 0.71 and R-squared = 0.66, respectively (S7 Fig). A semiquantitative interpretation of the hemoglobin results using WHO classifications resulted in an agreement of 87.6% on capillary and 93.6% on venous specimens (S6 and S7 Tables).

### Genetic association to enzyme activity

DNA sequencing of the complete G6PD coding sequence was conducted on specimens from 22 females and 13 males from the Pennsylvania and Washington studies. These were primarily selected to span the critical dynamic activity range. The results are tabulated in S8 Table. The results from the sequencing are summarized below:

- Four of four males with G6PD activity ≤ 30% had confirmed G6PD deficiency; two had the Mediterranean allele and two had the A- allele.
- One male with G6PD activity of 35% had an A- allele so was classified as normal by the reference assay, but corresponded to a hemizygous deficient male genotype. The STANDARD G6PD Test classified this sample as normal on the capillary specimen and as deficient on the venous specimen.
- Eight of eight males with G6PD activity ranging from 68% to 79% were confirmed as hemizygous normal.
- In all, 13 of 13 females with G6PD activity ranging from 46% to 75% were confirmed as heterozygous.
- One female with G6PD activity at 80% was also heterozygous (A-/wt).
- Eight of nine females with G6PD activity between 76% and 96% were confirmed as homozygous normal.

## Discussion

Testing for G6PD deficiency is typically only available in facilities with access to complex laboratory equipment [13]. The challenges in performing and accessing clinical G6PD testing are emphasized by the fact that despite the large number of moderate- and high-complexity clinical laboratories in the United States, only 47 participated in the G6PDS-A 2020 College of American Pathologists (CAP) proficiency survey for testing for G6PD deficiency. Some clinical indications for which knowledge of G6PD deficiency status would be beneficial, would greatly benefit from point-of-care testing for G6PD deficiency [30, 31]. One such example is *Plasmodium vivax* malaria case management to inform treatment with 8-aminoquinolines [32]. Kozenis, used to treat hypnozoite forms of *P*. *vivax* in infected individuals, has an established G6PD activity threshold > 70% for eligibility [5, 27, 28]. This study highlights the influence of blood disorders on the performance of a point-of-care test for G6PD deficiency, the STANDARD G6PD Test; the performance of the same test conducted at the point of care on non-anticoagulated capillary blood and venous K_2_EDTA blood across three sites in the United States; and the limitations of interlaboratory reference G6PD comparison, specifically normalization.

Through adaptation of a temperature-mediated G6PD abrogation method described recently [25], a contrived specimen panel covering the MDLs for G6PD activity, 30% and 70% activity, was developed and assessed across two laboratories, one on a manual spectrophotometer and the second on an automated chemistry analyzer but with the same G6PD enzyme reagent kit. Normalization of the G6PD activity into percent G6PD activity showed good correlation, but the accuracy across the two sets of values was not perfect. A previous meta-analysis showed the interlaboratory variability in hemoglobin normalized G6PD values (U/g Hb) [14]. The results from the contrived specimens showed that normalization into percent activity inherently contributes to inaccuracy in the G6PD reference testing and therefore case definitions. The contrived specimens were useful for generating blood specimens with G6PD activity around the MDLs, which are otherwise hard to enrich for through general prospective recruitment strategies. This approach provides an alternative to the chemical approach described previously, and is perhaps easier to implement [33]. Blinded testing of these samples on the STANDARD G6PD Test showed no false negatives and a good linear correlation with the reference assay results.

The performance of the STANDARD G6PD Test was evaluated at a specialized hematology laboratory in London, United Kingdom, on multiple common blood disorders and across a broad range of hemoglobin concentrations. Good correlation between the STANDARD G6PD Test and the reference assay for both G6PD activity and hemoglobin showed that STANDARD G6PD results were not differentially confounded by the blood disorders in comparison to the reference assays. For the 28 blood specimens with a WBC count of > 30 x 10^9^ cells/L included in the blood disorder study, it was possible to assess the impact of high WBC count on both the reference and point-of-care tests through depletion of the WBCs and measuring G6PD activity prior to and post depletion. Extremely high WBC counts significantly impact whole blood G6PD activity and can lead to false negatives, wherein individuals with G6PD deficiency may be classified as normal when the WBCs are not depleted [13]. Individuals with these levels of WBC counts are highly unlikely to present to outpatient clinics for malaria treatment or to blood donation centers, as these WBC levels are associated with severe disease.

Prospective clinical studies conducted at three sites in the United States (Florida, Pennsylvania, and Washington) were used to assess the clinical performance of the STANDARD G6PD Test on both non-anticoagulated fingerstick specimens and venous K_2_EDTA blood specimens. The STANDARD G6PD Test was run by health facility staff at the point of care. The STANDARD G6PD had good power to discriminate G6PD deficient males and females from normal males and intermediate and normal females, as well as females with intermediate G6PD activity from females with normal G6PD activity, as shown by the AUC in the ROC analysis and the sensitivity and specificity at the different MDLs. While the STANDARD G6PD Test performed equally well on both fingerstick and venous samples for diagnosing G6PD deficient cases (≤ 30% G6PD activity), the test performed slightly better on venous samples compared to fingerstick for the diagnosis of females with intermediate G6PD activity. This is possibly due in part to the inherent imprecision arising from fingerstick sampling, as shown in the fingerstick precision study presented here. Despite this, the study showed that all intermediate female samples falsely classified as normal had G6PD activity > 60%, previously considered as normal G6PD activity by WHO [34, 35]. The prevalence of deficient and intermediate cases in this study was within typical ranges for many countries, and the negative predictive power for mischaracterizing females with intermediate activity or less as normal was 98.8% (95% CI: 96.6–99.8) on fingerstick samples. A limitation of the clinical study was the limited number of cases with intermediate G6PD activity that were recruited.

The study focused on the diagnostic performance of the STANDARD G6PD Test on fingerstick specimens and for G6PD deficient cases (≤ 30%) and females with intermediate activity with a threshold at 70%. Historically, the most critical MDL for managing G6PD deficiency has been severe G6PD deficiency, and more recently the new antimalarial drug Kozenis is not recommended for individuals with < 70% G6PD activity [5, 36]. However, the current WHO Prequalification of In Vitro Diagnostics Programme guidelines recommend an 80% threshold for G6PD intermediate females [37]. The ROC analysis showed that for both fingerstick specimens and venous blood specimens, the AUC consistently dropped from the 70% threshold to the 80% threshold; as shown previously [38]. The results from this study identified (i) confirmed hemizygous males with G6PD activity as low as 68% and (ii) confirmed homozygous females with G6PD activity below 80%. These observations are supported by a recent large US based study correlating percent activity against reference range [39] and a study with females in Indonesia [40]. Establishing a performance threshold for intermediates at 80% G6PD activity will result in (i) a significant lowering of the positive predictive power of the test for G6PD intermediate cases due to the lower AUC and (ii) a significantly higher proportion of females with homozygous G6PD normal alleles included in the intermediate group, if they have equivalent activity distributions to hemizygous normal males. Even at 70% G6PD activity and with a high diagnostic performance, given the typically low prevalence rates for G6PD intermediate cases, the positive predictive power for heterozygous females with intermediate activity is low. While the test result can inform immediate medical decisions, there is a significant likelihood that females with an intermediate G6PD test result are genetically homozygous normal, and further testing would be required to confirm that females with intermediate G6PD activity have a G6PD deficient allele. This would also be the case with the reference assay, given the overlap in G6PD activity distribution between homozygous normal and heterozygous G6PD deficient females. The data presented here in conjunction with other emerging reports support a 70% threshold as a more appropriate threshold over 80% for defining normal females from a diagnostic perspective, which also coincides with that for Kozenis.

Interestingly, one hemizygous G6PD deficient male with the A- variant, known to be one of the less severe variants of G6PD deficiency, had 35% G6PD activity on the reference assay. While for the US studies, an exclusion criterion was self-reported blood transfusion within the last 90 days, a recent hemolytic event in this individual cannot be excluded. Similarly, higher than previously reported G6PD activity ranges for A- variants [41, 42] have also been reported by Powers et al. [43]. In the latter case, the specimens were not necessarily from healthy individuals, as these were cases referred by clinics to a reference laboratory.

The hemoglobin concentration results provided by the STANDARD G6PD Test were also evaluated against a reference assay in both the blood disorder study and the US clinical studies. The STANDARD G6PD Test result, showed a better performance venous blood than on capillary blood, although across all studies the overall percent agreement was > 87%. In low-resource settings where alternate or additional hemoglobin testing would not be available or necessarily conducted, the availability of the hemoglobin result is an additional benefit of the test.

In brief, the results of these studies support the use of the STANDARD G6PD Test at the point of care for screening for G6PD deficiency and females with intermediate G6PD activity. Additional studies in more diverse clinical settings are required to further understand the robustness of the test in a broader range of point-of-care settings.

## Supporting information

S1 Fig Workflows of the clinical evaluation studies. (A) Study conducted at Plasma MedResearch, Boca Raton, Florida, USA. Reference testing for G6PD was conducted at the PATH laboratories in Seattle, Washington, USA. (B) Studies conducted at Biological Specialty Company in Reading and Allentown, Pennsylvania, USA, and the Fred Hutchinson Cancer Research Center, Seattle, Washington, USA. Reference testing was conducted at the University of Washington Medical Center—Northwest clinical laboratory in Seattle.
(PDF)

S2 Fig Workflow of the contrived specimen study. Contrived specimens were prepared and split into three aliquots at the PATH laboratories (Seattle, Washington, USA). One aliquot was shipped to the Laboratory Alliance of Central New York, LLC (Syracuse, New York, USA), where the STANDARD G6PD Test was run. The reference assay was run on (i) a clinical chemistry analyzer at the University of Washington Medical Center—Northwest clinical laboratory (Seattle, Washington, USA) and (ii) a spectrophotometer at the PATH laboratories. All testing was blinded.
(PDF)

S3 Fig Correlation for hemoglobin values from the STANDARD G6PD Test and the reference values in the blood disorder study (London, United Kingdom). (PDF)

S4 Fig Bland-Altman plots to assess agreement between the STANDARD G6PD Test results and reference assay results in the blood disorder study (London, United Kingdom). (A) G6PD activity values (U/g Hb). (B) Hemoglobin concentration (g/dL).
(PDF)

S5 Fig Correlation between reference assays on contrived specimens. The reference G6PD assay values measured at the PATH laboratories (Seattle, Washington, USA) and the University of Washington Medical Center—Northwest clinical laboratory, also in Seattle, were normalized to their respective adjusted male median values. The plot shows the percent G6PD activity for each set of data.
(PDF)

S6 Fig Regression analysis of STANDARD G6PD Test G6PD activity. (A) Venous specimens and (B) capillary specimens compared to normalized spectrophotometric reference test values on venous specimens.
(PDF)

S7 Fig Regression analysis of STANDARD G6PD Test hemoglobin concentration values. (A) Venous specimens and (B) capillary specimens compared to reference test hemoglobin values on venous specimens.
(PDF)

S1 Table Inventory of confirmatory testing for the different blood conditions associated with specimens included in this study. (DOCX)

S2 Table G6PD status percent agreement in the UK blood disorder study. Thirty percent G6PD activity for both males and females and 70% for females were used as thresholds for the deficient and intermediate classifications, respectively, on the reference assay, and 4.0 U/g Hb and 6.0 U/g Hb were used for the same classifications on the STANDARD G6PD Test. Percent agreement: 98.2% (95% confidence interval: 87.1–96.5).
(DOCX)

S3 Table Anemia percent agreement in the UK blood disorder study. Percent agreement between the STANDARD G6PD Test and the reference assay hemoglobin status using World Health Organization anemia classifications.
(DOCX)

S4 Table Descriptive statistics for specimens with high white blood cell counts in the blood disorder study (London, United Kingdom). (DOCX)

S5 Table Percent agreement between the STANDARD G6PD Test and the spectrophotometric G6PD reference test. The manufacturer’s threshold values at 30% (4.0 U/g Hb) and 70% (6.0 U/g Hb) G6PD activity on venous K_2_EDTA blood samples. Percent agreement: 92.3% (95% confidence interval: 90.2–94.0).
(DOCX)

S6 Table Anemia percent agreement for the capillary specimens in the US studies. Percent agreement between the venous K_2_EDTA STANDARD G6PD Test and the reference assay for hemoglobin status using World Health Organization anemia classifications. Percent agreement: 87.6% (95% confidence interval: 84.7–94.1).
(DOCX)

S7 Table Anemia percent agreement for the venous specimens in the US studies. Percent agreement between the venous K_2_EDTA STANDARD G6PD Test and the reference assay for hemoglobin status using World Health Organization anemia classifications. Percent agreement: 93.6% (95% confidence interval: 91.4–95.4).
(DOCX)

S8 Table G6PD DNA sequencing results. (XLSX)

S1 Text White blood cell depletion protocol. (DOCX)

## Abbreviations

AUC: area under the curve
CBC: complete blood count
CI: confidence interval
CLIA: Clinical Laboratory Improvement Amendments
COVID-19: coronavirus disease 2019
FST: fluorescent spot test
G6PD: glucose-6-phosphate dehydrogenase
IRB: institutional review board
K_2_EDTA: ethylenediaminetetraacetic acid dipotassium salt dihydrate
MDL: medical decision limit
*P. vivax*: *Plasmodium vivax*
ROC: receiver operating characteristic
SARS-CoV-2: severe acute respiratory syndrome coronavirus 2
U/g Hb: units per gram of hemoglobin
UWMC-NW: University of Washington Medical Center—Northwest
WBC: white blood cell
WHO: World Health Organization
