# Supplementary material for: Reference and point-of-care testing for G6PD deficiency: Blood disorder interference, contrived specimens, and fingerstick equivalence and precision
Source: PLoS One. 2021 Sep 20;16(9):e0257560. doi: 10.1371/journal.pone.0257560 (PMC8452025; doi:10.1371/journal.pone.0257560)
Supplement: S1 Fig — (A) Study conducted at Plasma MedResearch, Boca Raton, Florida, USA. Reference testing for G6PD was conducted at the PATH laboratories in Seattle, Washington, USA. (B) Studies conducted at Biological Specialty Company in Reading and Allentown, Pennsylvania, USA, and the Fred Hutchinson Cancer Research Center, Seattle, Washington, USA. Reference testing was conducted at the University of Washington Medical Center—Northwest clinical laboratory in Seattle. (PDF) [file pone.0257560.s001.pdf]

S1A Fig

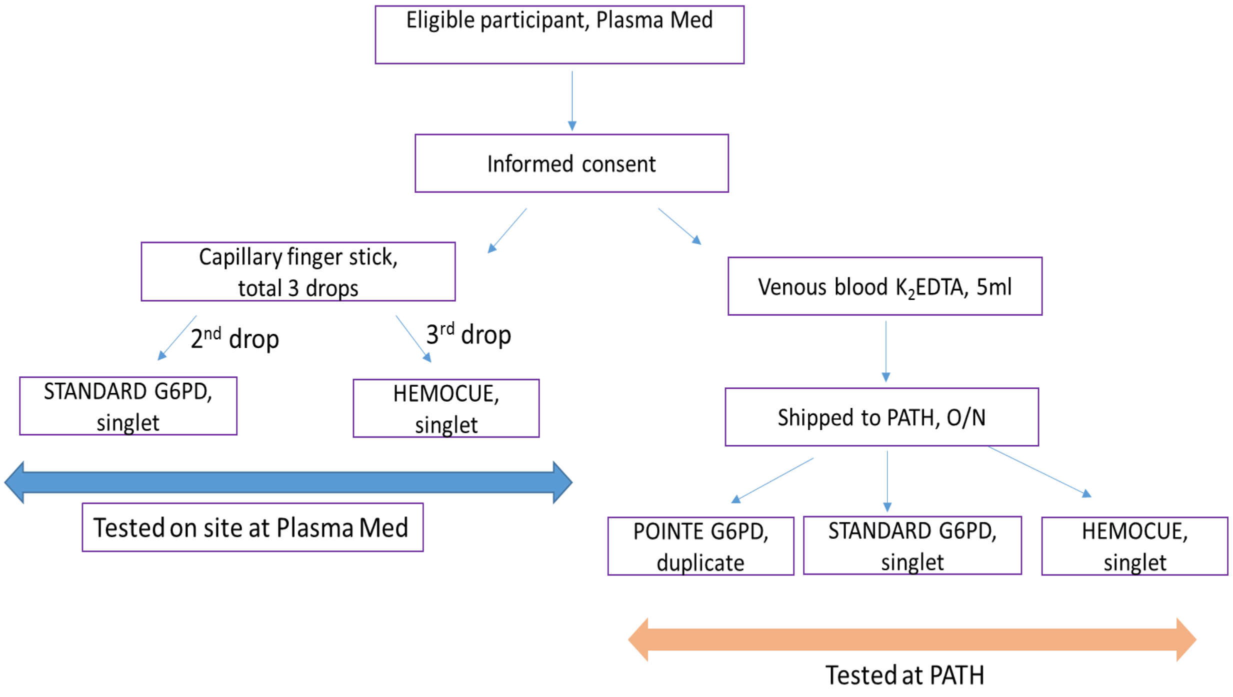

Abbreviations: EDTA, ethylenediaminetetraacetic acid; G6PD, glucose-6-phosphate dehydrogenase; O/N, overnight.

S1B Fig

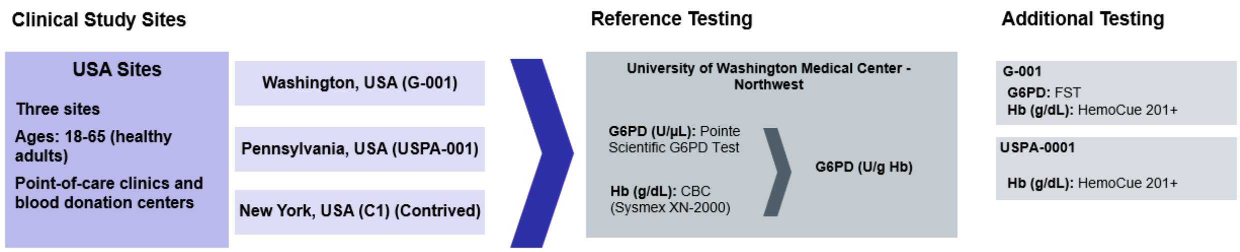

Abbreviations: CBC, complete blood count; FST, fluorescent spot test; G6PD, glucose-6-phosphate dehydrogenase; g/dL, grams per deciliter; Hb, hemoglobin; U/g Hb, units per gram of hemoglobin; U/µL, units per microliter.
