# Supplementary material for: Reference and point-of-care testing for G6PD deficiency: Blood disorder interference, contrived specimens, and fingerstick equivalence and precision
Source: PLoS One. 2021 Sep 20;16(9):e0257560. doi: 10.1371/journal.pone.0257560 (PMC8452025; doi:10.1371/journal.pone.0257560)
Supplement: S2 Fig — Contrived specimens were prepared and split into three aliquots at the PATH laboratories (Seattle, Washington, USA). One aliquot was shipped to the Laboratory Alliance of Central New York, LLC (Syracuse, New York, USA), where the STANDARD G6PD Test was run. The reference assay was run on (i) a clinical chemistry analyzer at the University of Washington Medical Center—Northwest clinical laboratory (Seattle, Washington, USA) and (ii) a spectrophotometer at the PATH laboratories. All testing was blinded. (PDF) [file pone.0257560.s002.pdf]

S2 Fig

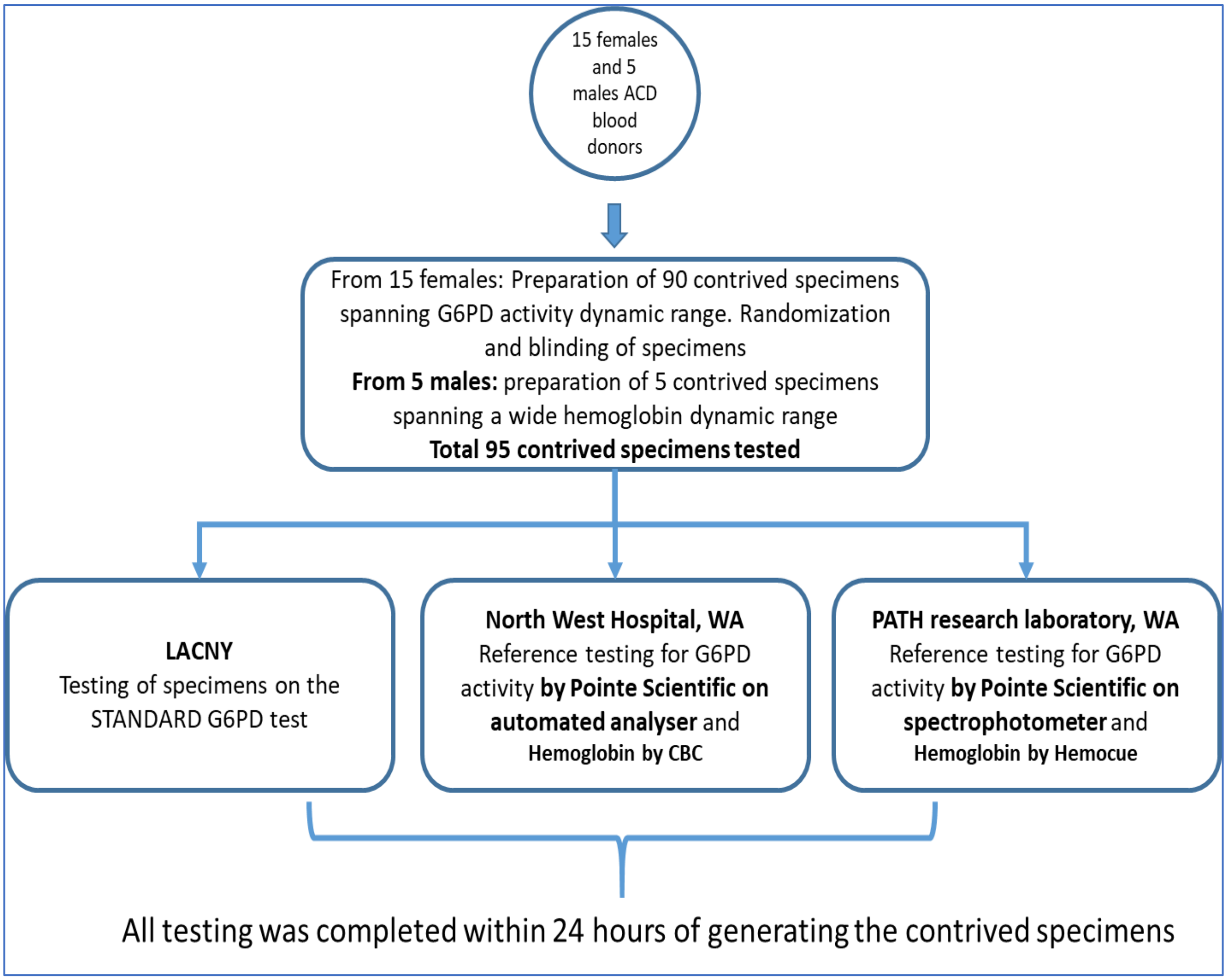

Abbreviations: ACD, acid citrate dextrose; CBC, complete blood count; G6PD, glucose-6-phosphate dehydrogenase; LACNY, Laboratory Alliance of Central New York, LLC; UWMC-NW, University of Washington Medical Center - Northwest clinical laboratory.
