# Supplementary material for: Reference and point-of-care testing for G6PD deficiency: Blood disorder interference, contrived specimens, and fingerstick equivalence and precision
Source: PLoS One. 2021 Sep 20;16(9):e0257560. doi: 10.1371/journal.pone.0257560 (PMC8452025; doi:10.1371/journal.pone.0257560)
Supplement: S4 Fig — (A) G6PD activity values (U/g Hb). (B) Hemoglobin concentration (g/dL). (PDF) [file pone.0257560.s004.pdf]

S4 Fig

(A) G6PD activity values (U/g Hb). (B) Hemoglobin concentration (g/dL).

A **B**

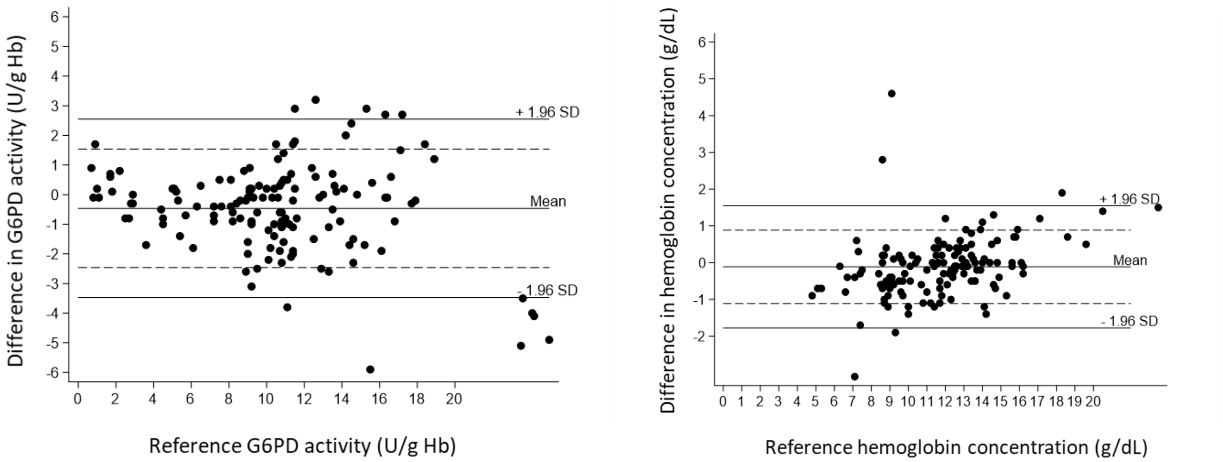

Abbreviations: G6PD, glucose-6-phosphate dehydrogenase; g/dL, grams per deciliter; U/g Hb, units per gram of hemoglobin.
