# Supplementary material for: Reference and point-of-care testing for G6PD deficiency: Blood disorder interference, contrived specimens, and fingerstick equivalence and precision
Source: PLoS One. 2021 Sep 20;16(9):e0257560. doi: 10.1371/journal.pone.0257560 (PMC8452025; doi:10.1371/journal.pone.0257560)
Supplement: S5 Fig — The reference G6PD assay values measured at the PATH laboratories (Seattle, Washington, USA) and the University of Washington Medical Center—Northwest clinical laboratory, also in Seattle, were normalized to their respective adjusted male median values. The plot shows the percent G6PD activity for each set of data. (PDF) [file pone.0257560.s005.pdf]

S5 Fig

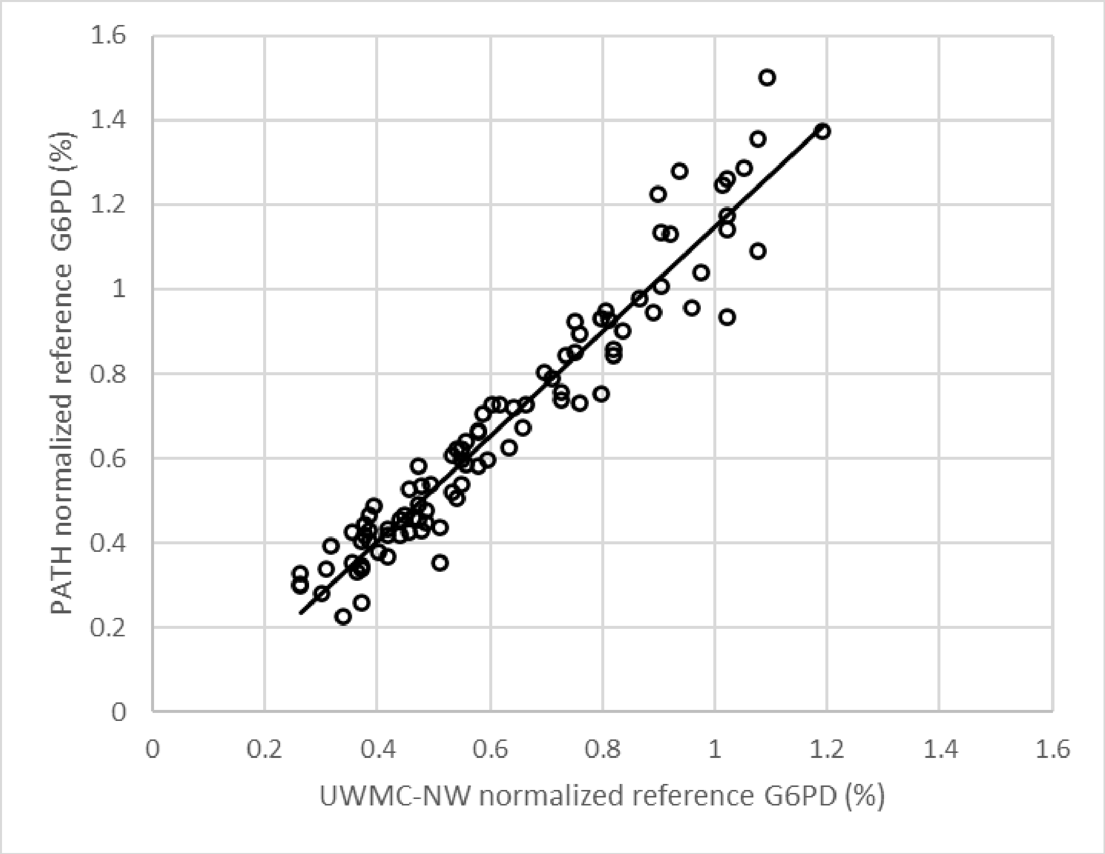

Abbreviations: G6PD, glucose-6-phosphate dehydrogenase; UWMC-NW, University of Washington Medical Center - Northwest clinical laboratory.
