# Supplementary material for: Reference and point-of-care testing for G6PD deficiency: Blood disorder interference, contrived specimens, and fingerstick equivalence and precision
Source: PLoS One. 2021 Sep 20;16(9):e0257560. doi: 10.1371/journal.pone.0257560 (PMC8452025; doi:10.1371/journal.pone.0257560)
Supplement: S7 Fig — (A) Venous specimens and (B) capillary specimens compared to reference test hemoglobin values on venous specimens. (PDF) [file pone.0257560.s007.pdf]

S8 Fig

A Venous

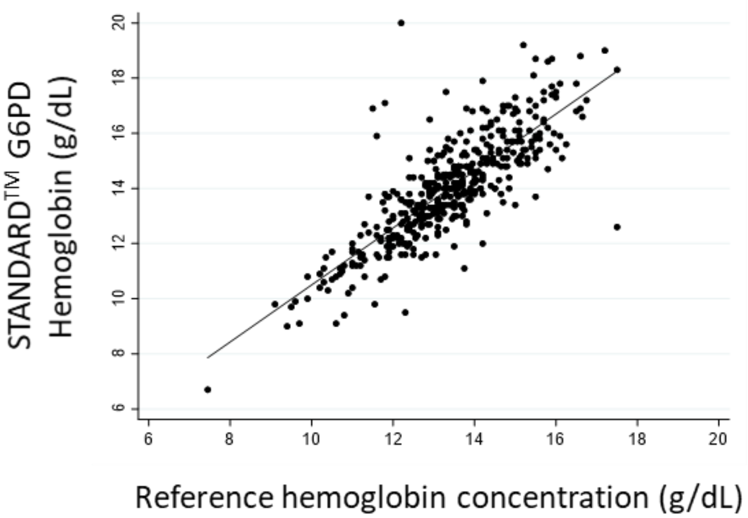

B Capillary

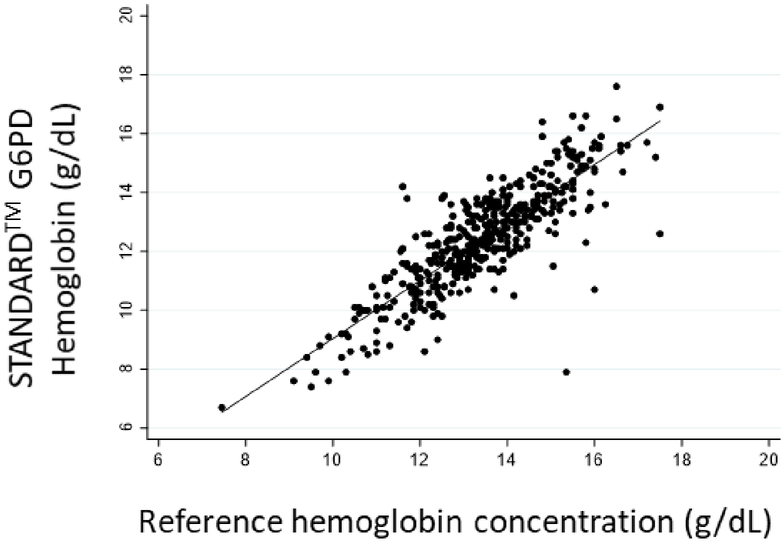

Abbreviations: G6PD, glucose-6-phosphate dehydrogenase; g/dL, grams per deciliter.
