# Supplementary material for: Reference and point-of-care testing for G6PD deficiency: Blood disorder interference, contrived specimens, and fingerstick equivalence and precision
Source: PLoS One. 2021 Sep 20;16(9):e0257560. doi: 10.1371/journal.pone.0257560 (PMC8452025; doi:10.1371/journal.pone.0257560)
Supplement: S1 Table — (DOCX) [file pone.0257560.s008.docx]

**Table S1.**

| **Assay** | **Laboratory** | **Profile** |
| --- | --- | --- |
| Cholesterol | Biochemistry | Routine lipid profile – Abbott ARCHITECT |
| Lipids (triglycerides) | Biochemistry | Routine lipid profile – Abbott ARCHITECT |
| Bilirubin | Biochemistry | Routine liver function tests – Abbott ARCHITECT |
| Protein (globulin) | Biochemistry | Routine – Abbott ARCHITECT |
| Glucose | Point-of-care testing/Biochemistry | GEM 500 blood gas analyzer/Abbott ARCHITECT |
| Lactic acid/Lactate | Point-of-care testing | GEM 500 blood gas analyzer |
| Lactate dehydrogenase | Biochemistry | Routine – Abbott ARCHITECT |
| Copper levels | Biochemistry trace metals | Agilent 7900 |
| Hematocrit/ Packed cell volume | Hematology | Full blood count – Abbott Alinity/Sysmex |
| Reticulocytes | Hematology | Full blood count – Abbott Alinity/Sysmex |
| White blood cell count | Hematology | Full blood count – Abbott Alinity/Sysmex |
| Platelets | Hematology | Full blood count – Abbott Alinity/Sysmex |
| Hemoglobin S | Special hematology | Bio-Rad Variant II and sickle screen |
| Hemoglobin D | Special hematology/DNA | Bio-Rad Variant II and DNA |
| Hemoglobin E | Special hematology | Bio-Rad Variant II and gel electrophoresis |
| Hemoglobin C | Special hematology | Bio-Rad Variant II and gel electrophoresis |
| Alpha thalassemia | Special hematology | Bio-Rad Variant II |
| Beta thalassemia | Special hematology | Bio-Rad Variant II |
| Iron levels | Biochemistry | Routine – Abbott ARCHITECT |
| Creatinine (renal function) | Biochemistry | Routine – Abbott ARCHITECT |
| Spherocytosis | Morphology | Microscopy |
| Platelet clumps | Hematology/Morphology | Full blood count and microscopy – Abbott Alinity/Sysmex |
| C-reactive protein | Biochemistry | Routine – Abbott ARCHITECT |
