# Supplementary material for: Reference and point-of-care testing for G6PD deficiency: Blood disorder interference, contrived specimens, and fingerstick equivalence and precision
Source: PLoS One. 2021 Sep 20;16(9):e0257560. doi: 10.1371/journal.pone.0257560 (PMC8452025; doi:10.1371/journal.pone.0257560)
Supplement: S2 Table — Thirty percent G6PD activity for both males and females and 70% for females were used as thresholds for the deficient and intermediate classifications, respectively, on the reference assay, and 4.0 U/g Hb and 6.0 U/g Hb were used for the same classifications on the STANDARD G6PD Test. Percent agreement: 98.2% (95% confidence interval: 87.1–96.5). (DOCX) [file pone.0257560.s009.docx]

**Table S2**

|  | | **Reference assay** | | | |
| --- | --- | --- | --- | --- | --- |
|  |  | Deficient | Intermediate | Normal | Total |
| **STANDARD G6PD Test** | Deficient | 10 | 5 | 4 | 19 |
|  | Intermediate | 0 | 6 | 0 | 6 |
|  | Normal | 0 | 1 | 112 | 113 |
|  | Total | 10 | 12 | 116 | 138 |

Abbreviation: G6PD, glucose-6-phosphate dehydrogenase.
