# Supplementary material for: Reference and point-of-care testing for G6PD deficiency: Blood disorder interference, contrived specimens, and fingerstick equivalence and precision
Source: PLoS One. 2021 Sep 20;16(9):e0257560. doi: 10.1371/journal.pone.0257560 (PMC8452025; doi:10.1371/journal.pone.0257560)
Supplement: S3 Table — Percent agreement between the STANDARD G6PD Test and the reference assay hemoglobin status using World Health Organization anemia classifications. (DOCX) [file pone.0257560.s010.docx]

**Table S3**

| **Agreement for classification of anemia including all samples; percent agreement:** **88% (95% CI: 82.1–92.5)** | | **Classification by reference assay** | | | |
| --- | --- | --- | --- | --- | --- |
|  |  | Non/Mild anemia | Moderate anemia | Severe anemia | Total |
| **Classification by STANDARD G6PD Test** | Non/Mild anemia | 90 | 3 | 0 | 93 |
|  | Moderate anemia | 8 | 42 | 0 | 50 |
|  | Severe anemia | 0 | 9 | 15 | 24 |
|  | Total | 98 | 54 | 15 | 167 |
| **Agreement for classification of anemia excluding high white blood cell count; percent agreement 89.9% (95% CI: 83.6–94.3)** | | **Classification by reference assay** | | | |
|  |  | Non/Mild anemia | Moderate anemia | Severe anemia | Total |
| **Classification by STANDARD G6PD Test** | Non/Mild anemia | 77 | 2 | 0 | 79 |
|  | Moderate anemia | 6 | 33 | 0 | 39 |
|  | Severe anemia | 0 | 6 | 14 | 20 |
|  | Total | 83 | 41 | 14 | 138 |

Abbreviations: CI, confidence interval; G6PD, glucose-6-phosphate dehydrogenase.
