# Supplementary material for: Reference and point-of-care testing for G6PD deficiency: Blood disorder interference, contrived specimens, and fingerstick equivalence and precision
Source: PLoS One. 2021 Sep 20;16(9):e0257560. doi: 10.1371/journal.pone.0257560 (PMC8452025; doi:10.1371/journal.pone.0257560)
Supplement: S4 Table — (DOCX) [file pone.0257560.s011.docx]

**Table S4**

| **Number of matched samples** | 28 | | | |
| --- | --- | --- | --- | --- |
| **Males** | 15 | | | |
| **Females** | 13 | | | |
| **WBC whole blood** | | | | |
| Range | 30–392 x 10^9^ cells/L | | | |
| Mean | 77.4 x 10^9^ cells/L | | | |
| Median | 56 x 10^9^ cells/L | | | |
| **WBC-depleted blood** | | | | |
| Range | 0.2–94.6 x 10^9^ cells/L | | | |
| Mean | 9.28 x 10^9^ cells/L | | | |
| Median | 4.07 x 10^9^ cells/L | | | |
|  | | | | |
|  | **Hb-normalized Pointe Scientific G6PD results** | | **STANDARD G6PD Test results (run 1)** | |
|  | **Whole blood** | **Depleted blood** | **Whole blood** | **Depleted blood** |
| **G6PD range** | 3.5–22.4 | 0.8–17.7 | 4.4–20.1 | 1.2–20.1 |
| **Mean G6PD value** | 12.9 | 8.7 | 13.6 | 8.7 |
| **Median G6PD value** | 12.8 | 8.2 | 13.1 | 7.85 |
| **Hb range** | 7.4–15.4 | 7.4–14.1 | 6.6–17.5 | 5.8–14.6 |
| **Mean Hb value** | 10.9 | 11.3 | 10.89 | 10.7 |
| **Median Hb value** | 11.0 | 11.55 | 10.85 | 11.4 |
| **Deficient G6PD** | 0 | 2 | 0 | 2 |

Abbreviations: G6PD, glucose-6-phosphate dehydrogenase; Hb, hemoglobin; WBC, white blood cell.

* The same four samples in both STANDARD G6PD Test undepleted runs produced a “Hi” G6PD value on the test reader. For purposes of the descriptive analysis, these were replaced with 20.1 IU/g Hb (international units per gram of hemoglobin). Two samples in run 1 of the white blood cell–depleted samples produced a “Hi” read for G6PD; this was also replaced with 20.1 IU/g Hb.
