# Supplementary material for: Reference and point-of-care testing for G6PD deficiency: Blood disorder interference, contrived specimens, and fingerstick equivalence and precision
Source: PLoS One. 2021 Sep 20;16(9):e0257560. doi: 10.1371/journal.pone.0257560 (PMC8452025; doi:10.1371/journal.pone.0257560)
Supplement: S5 Table — The manufacturer’s threshold values at 30% (4.0 U/g Hb) and 70% (6.0 U/g Hb) G6PD activity on venous K2EDTA blood samples. Percent agreement: 92.3% (95% confidence interval: 90.2–94.0). (DOCX) [file pone.0257560.s012.docx]

**Table S5**

|  | | **Spectrophotometric reference test** | | | |
| --- | --- | --- | --- | --- | --- |
|  |  | Deficient | Intermediate | Normal | Total |
| **STANDARD G6PD Test** | Deficient | 56 | 11 | 15 | 82 |
|  | Intermediate | 0 | 15 | 34 | 49 |
|  | Normal | 0 | 1 | 658 | 659 |
|  | Total | 56 | 27 | 707 | 790 |
