# Supplementary material for: Reference and point-of-care testing for G6PD deficiency: Blood disorder interference, contrived specimens, and fingerstick equivalence and precision
Source: PLoS One. 2021 Sep 20;16(9):e0257560. doi: 10.1371/journal.pone.0257560 (PMC8452025; doi:10.1371/journal.pone.0257560)
Supplement: S6 Table — Percent agreement between the venous K2EDTA STANDARD G6PD Test and the reference assay for hemoglobin status using World Health Organization anemia classifications. Percent agreement: 87.6% (95% confidence interval: 84.7–94.1). (DOCX) [file pone.0257560.s013.docx]

**Table S6**

| **Agreement for classification of anemia: capillary specimens** | | **Classification by reference assay** | | | |
| --- | --- | --- | --- | --- | --- |
|  |  | Non/Mild anemia | Moderate anemia | Severe anemia | Total |
| **Classification by STANDARD G6PD Test** | Non/Mild anemia | 499 | 3 | 0 | 502 |
|  | Moderate anemia | 67 | 43 | 0 | 110 |
|  | Severe anemia | 1 | 6 | 2 | 9 |
|  | Total | 567 | 52 | 2 | 621 |

Abbreviation: G6PD, glucose-6-phosphate dehydrogenase.
