# Supplementary material for: Reference and point-of-care testing for G6PD deficiency: Blood disorder interference, contrived specimens, and fingerstick equivalence and precision
Source: PLoS One. 2021 Sep 20;16(9):e0257560. doi: 10.1371/journal.pone.0257560 (PMC8452025; doi:10.1371/journal.pone.0257560)
Supplement: S7 Table — Percent agreement between the venous K2EDTA STANDARD G6PD Test and the reference assay for hemoglobin status using World Health Organization anemia classifications. Percent agreement: 93.6% (95% confidence interval: 91.4–95.4). (DOCX) [file pone.0257560.s014.docx]

**Table S7**

| **Agreement for classification of anemia: venous K_2_EDTA specimens** | | **Classification by reference assay** | | | |
| --- | --- | --- | --- | --- | --- |
|  |  | Non/Mild anemia | Moderate anemia | Severe anemia | Total |
| **Classification by STANDARD G6PD Test** | Non/Mild anemia | 528 | 6 | 0 | 534 |
|  | Moderate anemia | 31 | 44 | 0 | 75 |
|  | Severe anemia | 0 | 2 | 2 | 4 |
|  | Total | 559 | 52 | 2 | 613 |

Abbreviations: K_2_EDTA, ethylenediaminetetraacetic acid dipotassium salt dihydrate; G6PD, glucose-6-phosphate dehydrogenase.
