# Supplementary material for: Reference and point-of-care testing for G6PD deficiency: Blood disorder interference, contrived specimens, and fingerstick equivalence and precision
Source: PLoS One. 2021 Sep 20;16(9):e0257560. doi: 10.1371/journal.pone.0257560 (PMC8452025; doi:10.1371/journal.pone.0257560)
Supplement: S1 Text — (DOCX) [file pone.0257560.s016.docx]

***White blood cell depletion protocol outline****:*

- *A pure red cell suspension is prepared by removing leucocytes and platelets.*
- *Remember to always include a normal control.*
- *Pure red cell suspensions can be made from whole blood by filtering the blood through a mixed bed of microcrystalline cellulose and alpha cellulose.*
- *Approximately 0.5 g of each type of cellulose is mixed with 20 ml of saline which gives sufficient slurry for 2 columns.*
- *The barrel of a 5 ml syringe is used as a column.*
- *The outlet of the syringe is blocked with absorbent cotton wool, equal in volume to the 1ml mark on the barrel. Tease the wool out then lightly pack to the 1 ml mark.*
- *The well-shaken slurry is pipetted into the column to give a bed volume of 2 ml after the saline has run through.*
- *The bed is washed with 5 ml of saline to remove any “fines”.*
- *When the saline has run through discard any collected liquid and then add 1 ml of whole blood carefully onto the column and collect the filtrate in a large centrifuge tube. Once the blood has completely run into the bed, wash through with 4 – 5 ml of saline.*
- *Once the filtered red cells have been collected, centrifuge and make a 50/50 suspension with saline and run an FBC on this to check the WBC of the filtered sample to ensure that all white cells and platelets have been removed. By this method >95% of the WBC should be removed.*
- *The procedure should not alter the age or size of distribution of the recovered red cells compared to native blood. This should be checked with each new batch of cellulose by counting reticulocytes.*
- *Conduct whole blood cell count to confirm white blood cell depletion and measure hemoglobin concentration*
- *Conduct G6PD assay*
